# Host Factors Determine Anti-GM1 Response Following Oral Challenge of Chickens with Guillain-Barré Syndrome Derived *Campylobacter jejuni* Strain GB11

**DOI:** 10.1371/journal.pone.0009820

**Published:** 2010-03-22

**Authors:** C. Wim Ang, Jeroen R. Dijkstra, Marcel A. de Klerk, Hubert Ph. Endtz, Pieter A. van Doorn, Bart C. Jacobs, Suzan H. M. Jeurissen, Jaap A. Wagenaar

**Affiliations:** 1 Department of Medical Microbiology and Infection Control, VU University Medical Center, Amsterdam, The Netherlands; 2 Department of Neurology, Erasmus University Medical Centre, Rotterdam, The Netherlands; 3 Department of Immunology, Erasmus University Medical Centre, Rotterdam, The Netherlands; 4 Department of Medical Microbiology and Infectious Diseases, Erasmus University Medical Centre, Rotterdam, The Netherlands; 5 International Centre for Diarrhoeal Disease Research, Dhaka, Bangladesh; 6 Central Veterinary Institute of Wageningen UR, Lelystad, The Netherlands; 7 Department of Infectious Diseases and Immunology, Faculty of Veterinary Medicine, Utrecht University, Utrecht, The Netherlands; 8 WHO Collaborating Center for Campylobacter/OIE Reference Laboratory for Campylobacteriosis, Utrecht, The Netherlands; Charité-Universitätsmedizin Berlin, Germany

## Abstract

**Background:**

Anti-ganglioside antibodies with a pathogenic potential are present in *C. jejuni*-associated Guillain-Barré syndrome (GBS) patients and are probably induced by molecular mimicry. Immunization studies in rabbits and mice have demonstrated that these anti-ganglioside antibodies can be induced using purified lipo-oligosaccharides (LOS) from *C. jejuni* in a strong adjuvant.

**Methodology/Principal Findings:**

To investigate whether natural colonization of chickens with a ganglioside-mimicking *C. jejuni* strain induces an anti-ganglioside response, and to investigate the diversity in anti-ganglioside response between and within genetically different chicken lines, we orally challenged chickens with different *C. jejuni* strains. Oral challenge of chickens with a *C. jejuni* strain from a GBS patient, containing a LOS that mimics ganglioside GM1, induced specific IgM and IgG anti-LOS and anti-GM1 antibodies. Inoculation of chickens with the Penner HS:3 serostrain, without a GM1-like structure, induced anti-LOS but no anti-ganglioside antibodies. We observed different patterns of anti-LOS/ganglioside response between and within the five strains of chickens.

**Conclusions:**

Natural infection of chickens with C. jejuni induces anti-ganglioside antibodies. The production of antibodies is governed by both microbial and host factors.

## Introduction

Infections in humans with the enteric pathogen *Campylobacter jejuni* are able to trigger neurologic sequelae such as the Guillain-Barré (GBS) and Miller Fisher syndrome (MFS) [1]. Neurologic symptoms are mediated by complement binding anti-ganglioside antibodies [2]. These anti-ganglioside antibodies cross-react with the lipo-oligosaccharide (LOS) fraction of GBS-associated *C. jejuni* strains and are therefore presumed to be induced by molecular mimicry [3].

Immunization of various animal species with purified preparations of ganglioside-mimicking *C. jejuni* LOS leads to an anti-ganglioside response, thereby validating the molecular mimicry hypothesis [4], [5], [6]. However, the efficiency of the immunization procedure is heavily dependent on the use of strong adjuvants, unlike the situation in GBS and MFS patients, where the anti-ganglioside antibody response is induced after a natural infection with *C. jejuni*. Previous studies have reported the induction of anti-ganglioside antibodies in chickens following *C. jejuni* infection but these have been performed with *C. jejuni* strains with an uncharacterized LOS fraction and without non-ganglioside mimicking control *C. jejuni* strain [7], [8]. A single unconfirmed study reported the presence of a GBS-like disease in chickens following inoculation with a *C. jejuni* strain [9].

Only a very small proportion of *C. jejuni* infected human individuals develop GBS or MFS. Bacterial risk factors for the development of neurological disease identified so far, are genes or gene polymorphisms located within the LOS gene cluster, emphasizing the important role of LOS in the pathogenesis of *Campylobacter*-related GBS [10], [11]. However, family studies indicate that although multiple individuals can be infected by the same *Campylobacter* strain, not all family members develop GBS [12]. These observations suggest that, in addition to bacterial traits, host-determined factors play a role in the development of post-*Campylobacter* neuropathy.

In the present study, we performed a series of experiments where we orally challenged several groups of chickens with GBS-associated strain GB11 in order to answer the following questions. (i) Is it possible to induce an anti-ganglioside antibody response in chickens by a natural route of inoculation, as opposed to the non-physiological adjuvant-dependent immunizations. (ii) Is there any difference in anti-ganglioside response either between or within genetically different groups of chickens.

## Materials and Methods

### Animals

Five genetically different meat-type chicken groups were used for this experiment. They included two traditional Old Dutch Breeds, groups 1 (Barnevelder) and 2 (Twentsche Grijzen), obtained from IPC dier, Barneveld, the Netherlands. Three modern outbred broiler groups were included, groups E3 (meat-type), E4 (meat-type but also selected for reproduction) and E5 (offspring of group E3×group E4 cross) Groups 3, 4, and 5 were kindly provided by Euribrid (Herveld, The Netherlands). After hatch, birds were tested and shown to be free of *Campylobacter*. Birds were given feed and water ad libitum. All chickens were cared for in accordance with accepted procedures of the Dutch law of animal welfare. The animal experiment committee (DEC, Dier Experimentele Commissie) from the Central Veterinary Institute approved all experimental procedures applied to the birds (ID-Lelystad/CVI, project number 367.47026.00). All experiments were performed in isolators with an absolute separation of the different groups.

### Bacterial strains

*C. jejuni* strain GB11 was isolated from a patient with GBS with anti-GM1, anti-GD1b and anti-GA1 antibodies [13], [14]. The LOS structure has been determined previously and was shown to contain GM1 and GD1a mimics [14]. As a control, the Penner HS:3 serostrain was used. This LOS of this strain does not mimic any ganglioside [15]. *C. jejuni* strains were grown on blood agar plates, incubated at 37°C under microaerobic conditions.

### Inoculation experiments

Chickens were orally challenged at day 15 after hatch with 10*9 cfu of bacteria in 0.5 ml by oral gavage. Per chicken group, 5–12 animals were used. Control animals were orally challenged with phosphate-buffered saline (PBS). Birds were observed daily for the development of neurological symptoms. Blood sampling was performed on days 0, 8, 16, and 21. At day 27 post-inoculation, the animals were sacrificed, an additional blood sample was taken and caecal contents were sampled for culture of *C. jejuni*. Samples from sciatic nerves of selected animals were fixed in glutaraldehyde, post-fixed with osmium tetroxide and embedded in epoxy resin. Semithin sections were stained with toluidine blue and investigated with light microscopy. For electron microscopy, ultrathin sections were stained with uranyl acetate and lead citrate before examination.

### Serological studies

Serum samples were investigated for antibody reactivity against *C. jejuni* protein fractions, purified *C. jejuni* LOS and purified gangliosides using ELISA. For anti-protein reactivity, *C. jejuni* proteins were extracted with acid glycine. ELISA plates (Maxisorp, NUNC, Roskilde, Denmark) were coated overnight with 5 microgram/ml protein per well in Na_2_CO_3_ buffer pH 9.6. Serum samples were diluted 1∶100 in PBS and incubated at 37°C for 1 hour. After washing with PBS containing 0.05% Tween20 (PBS-Tween) plates were incubated with monoclonal mouse anti-chicken IgM (CVI-ChIgG 59.7) or IgG (CVI-ChIgM 47.3) diluted 1∶10,000 or 1∶7,500 in PBS at 37°C for 1 hour. Plates were washed with PBS-Tween and incubated with anti-mouse total immunoglobulines conjugated to peroxidase (Dako, Glostrup, Denmark) diluted 1∶1,000 at 37°C for 1 hour. Plates were developed with o-phenylenediamine, the reaction was stopped by adding 2N HCl and plates were read at 490 nm.

The LOS fraction of *C. jejuni* was extracted with hot phenol-water [16]. Purified freeze dried fractions were weighed and resuspended in water. For detection of antibodies against *C. jejuni* LOS, plates were coated overnight with 1 microgram LOS/well in PBS as described previously [17]. ELISA was performed as stated above for the detection of anti- *C. jejuni* protein antibodies with the following modifications. Serum incubation was performed at 4°C overnight, other incubations were also at 4°C. Washing was done with PBS without Tween. For anti-ganglioside reactivity, plates were coated with asialo-GM1 (GA1), GM1, GD1b or GQ1b as described before [17] and anti-ganglioside antibody reactivity was detected as described above for anti-LOS reactivity.

To assess cross-reactivity, serum from infected chickens was incubated with *Campylobacter* LOS conjugated to Octyl-Sepharose CL4b beads as described previously [3] and absorbed serum samples were tested for anti-LOS and anti-ganglioside reactivity.

## Results

### Specificity of the antibody response

**Figure 1:**
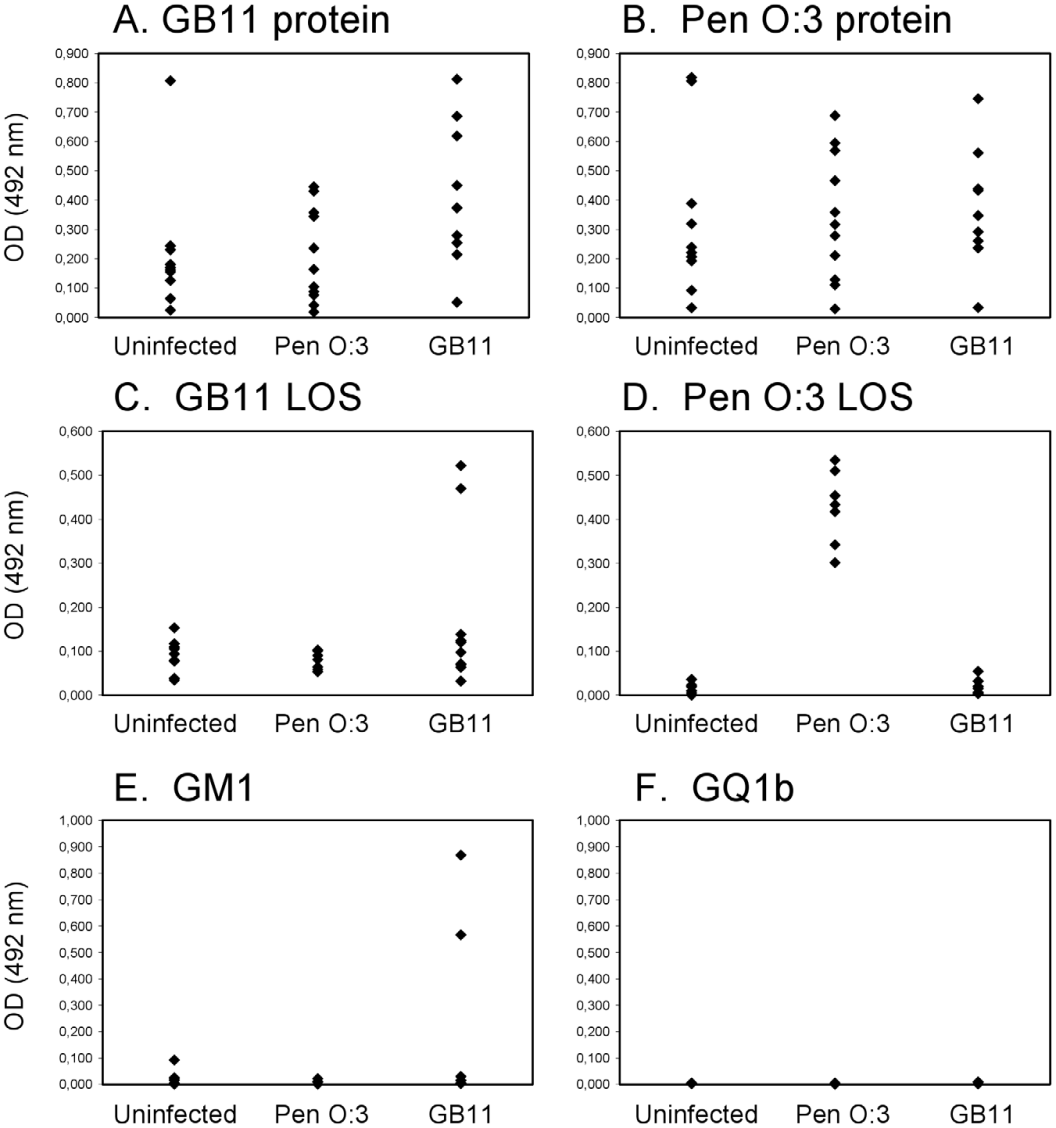
IgG antibody responses against *C. jejuni* antigens. IgG reactivity against *C. jejuni* proteins (A,B), lipo-oligosaccharides (LOS) (C,D) and gangliosides GM1 and GQ1b (E,F) in E4 chickens inoculated with *C. jejuni* strains Pen HS:3 and GB11. Serum was collected at day 27 post-infection. Each dot represents one chicken.

For determination of the specificity of the antibody response following colonization with *Campylobacter*, chickens from group E4 were orally challenged with GB11, Pen HS:3 or PBS and the antibody response against proteins, LOS and gangliosides was determined at 27 days post-inoculation. *Campylobacter*-challenged chickens had widely divergent antibody responses against protein antigens (Fig. 1A, B). There was both an IgM and IgG response against the protein antigen, but the IgM response was generally lower than the IgG response (data not shown). In GB11 challenged chickens, the IgG antibody response against GB11 protein was slightly stronger than against Pen HS:3 protein (Fig. 1A), but generally, there was considerable cross-reactivity between groups of chickens. In contrast, the IgG anti-LOS responses were highly specific. All Pen HS:3 infected chickens had a strong IgG anti-Pen HS:3 LOS response, but none of the animals had reactivity against GB11 LOS (Fig. 1C). Only 2/9 GB11 challenged chickens mounted a strong IgG response against GB11 LOS (Fig. 1C). This reactivity was highly specific because these two sera did not have reactivity against Pen HS:3 LOS.

The anti-ganglioside reactivity in chickens was as would be expected from the ganglioside-structure of the infecting *Campylobacter* strain. There was no detectable anti-GM1 reactivity in chickens that were not orally challenged or chickens that had been orally challenged with Pen HS:3 (Fig. 1E) The two GB11 challenged chickens with an anti-LPS response also had an anti-ganglioside response against GA1, GM1 and GD1b, but not against GQ1b (Fig. 1E, F and data not shown). This pattern of anti-ganglioside reactivity was identical to that of the patient from which strain GB11 was isolated. We tested whether the anti-ganglioside antibodies were indeed induced by molecular mimicry by absorbing the anti-ganglioside and anti-LOS antibodies with *C. jejuni* LOS conjugated to Octyl-sepharose beads. The anti-GM1 and anti-GB11 LOS reactivity could be reduced by 60–90% following incubation with GB11-coupled sepharose beads but not by Pen HS:3 coupled beads or beads without LOS, thereby demonstrating that the antibodies are cross-reactive (data not shown).

### Difference between chicken lines

**Figure 2:**
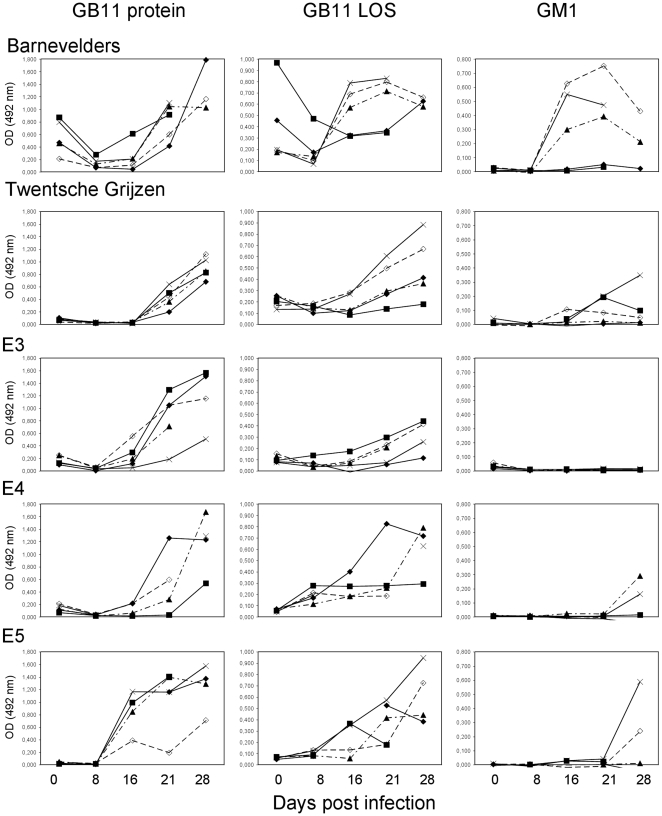
Kinetics of IgG antibody responses against *C. jejuni* antigens. IgG reactivity against *C. jejuni* GB11 protein, lipo-oligosaccharide (LOS) and ganglioside GM1 in five groups of chickens inoculated with *C. jejuni* GB11. Serum was collected at days 0, 8, 16, 21 and 27 post-infection. Each line represents one chicken. Serum was not available for all time points for all chickens.

To investigate the influence of genetic background on the ability to mount an anti-*C. jejuni* antibody response, we orally challenged five chicken lines with *C. jejuni* strain GB11. Again we found a strong anti-protein response in all orally challenged chickens but there were remarkable differences between the groups (Fig. 2). In serum samples taken before inoculation, there was already a moderate response against GB11 protein in the Barnevelders but not in the other groups. This was probably due to passively transferred maternal antibodies because IgG anti-GB11 protein levels decreased one week post inoculation to rise again between two and three weeks post inoculation. The IgG anti-protein response in E5 chickens appeared one week earlier than for the other chicken lines. For IgM anti-protein responses, again the Barnevelders had a different pattern of response. This group showed slowly increasing IgM anti-protein levels whereas in the other groups the IgM levels rose steeply in the second week following inoculation and declined after three weeks (data not shown).

The IgG anti-LOS response was stronger in three of five individual Barnevelders and appeared earlier than in the other groups. In most Barnevelders, there was a strong IgG anti-GB11 LOS response detectable two weeks post-inoculation, whereas in the other groups, the anti-LOS response lagged one to two weeks (Fig 2). In addition to detectable anti-protein responses before oral challenge, two out of five of the Barnevelders also had detectable anti-LOS reactivity in the pre-infection samples. Interestingly, the chickens with the highest pre-challenge anti-LOS antibody level, had only modest responses post-infection. Similar to the anti-protein antibodies, the anti-LOS antibody level also decreased in the first week after infection and increased subsequently.

The anti-ganglioside reactivity also showed a differential pattern between groups of chickens. The Barnevelders had the earliest detectable anti-GM1 antibodies and also the proportion of chickens with an anti-ganglioside response was higher in the Barnevelders than in the other animals (Fig. 2). None of the chickens of the E3 group had detectable IgG anti-GM1 reactivity. The Barnevelders and Twentsche Grijzen were the only groups with a substantial IgM anti-GM1 response (data not shown).

### Difference within chicken lines

In addition to a widely divergent antibody response to *Campylobacter* antigens and gangliosides between chicken lines, we also observed large differences within chicken lines. In all experiments, the antibody response varied widely within chicken lines, irrespective of the type of antigen (Fig 1,2). This ranged from the complete absence of IgG antibody reactivity against GM1 in seven out of nine GB11 challenged E4 chickens (Fig 1E) to a continuous range of antibody reactivity against protein antigens in several groups of chickens (Fig 1A,B, Fig 2).

In one experiment, the chickens developed clinical symptoms compatible with damage to peripheral nerves. The chickens had weakness of legs and wings. The attack rate was 1/5 in the E3 group, 3/5 in the E4 group and 3/5 in the E5 group. However, histopathological analysis of sciatic nerves of these chickens did not reveal any abnormalities, especially no demyelination or axonal degeneration. Furthermore, there was no relation between presence and magnitude of the anti-ganglioside response and the presence of neurological symptoms. Additional testing for infection with Marek's disease virus in these chickens was negative.

**Figure 3:**
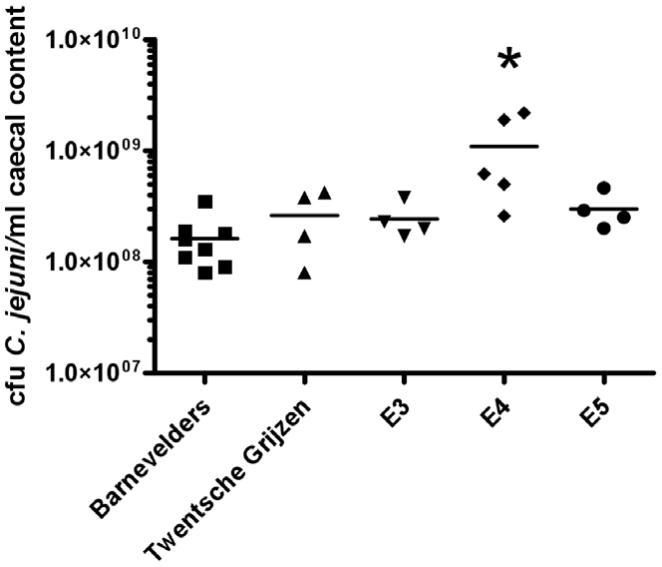
Germ counts in caecal contents of five groups of chickens inoculated with *C. jejuni* GB11. Bars indicate mean cfu/per group. * p-value 0.003 (Mann-Whitney U, E4 compared to all other groups).

Quantitative culture of caecal contents for *C. jejuni* revealed that the E4 chickens had the highest colonization level (Fig 3). When data from all chickens were analyzed together, there was no relation between colonization level and magnitude of the antibody response against protein or glycolipid antigens (LOS and gangliosides). However, within the E4 and E5 groups, *C. jejuni* counts in caecal content were highest in chickens with the strongest antibody response against glycolipid antigens (data not shown).

## Discussion

We demonstrated that chickens mount a cross-reactive anti-*Campylobacter* LOS/ganglioside response following natural colonization with a ganglioside mimicking *C. jejuni* strain from a GBS patient. The anti-ganglioside response was specific and the kinetics and strength of the anti-ganglioside response differed between and within groups of genetically diverse chickens.

The specificity of the anti-ganglioside response in chickens was similar to that in the patient from which GB11 was cultured. In addition, absorption studies demonstrated cross-reactivity between anti-LOS and anti-ganglioside antibodies, indicating that in both chickens and GBS patient, the antibodies were induced by molecular mimicry. The kinetics of the anti-ganglioside response in chickens was also similar to that observed in GBS patients. In both chickens and GBS patients, the anti-ganglioside responses peak between two and four weeks following inoculation with *C. jejuni*. No anti-ganglioside antibodies were formed following inoculation with Pen HS:3, although in chickens infected with Pen HS:3, there was a strong antibody response against both Pen HS:3 protein and LOS. Together with the consistent absence of anti-GM1 response in PBS and HS:3 orally challenged animals, it can be concluded that the anti-ganglioside antibodies are specifically induced by inoculation with a ganglioside-mimicking *C. jejuni* strain. Immunization studies of mice and rabbits with *Campylobacter* LOS in strong adjuvants reached similar conclusions and our results are in accordance with these studies [3], [4], [6].

Usuki et al. described the induction of anti-GM1 antibodies in chickens following inoculation with multiple *C. jejuni* strains, including strain 81116 [8]. The exact LOS structure of these strains has not been determined but it is known that *C. jejuni* 81116 does not express a GM1 structure [18]. Surprisingly, Usuki et al. describe the presence of anti-GM1 antibodies following inoculation with *C. jejuni* 81116 [8]. The authors did not perform any absorption studies and therefore it remains unclear whether the anti-ganglioside antibodies that were detected in their study are truly cross-reactive with *C. jejuni* LOS.

We observed differences between chicken strains in frequency of development of anti-ganglioside antibodies, the kinetics of the antibody response, and colonization following inoculation of animals with strain GB11. Although the numbers of animals per group were small and there was variation within each group, we think that our data clearly demonstrate that, in addition to bacterial properties, host derived factors influence the anti-ganglioside response. The first possible explanation is the presence of genetically based differences between the groups of chickens in handling and processing of bacterial antigens by the immune system [19]. Experiments investigating disease resistance of Salmonella found that Barnevelders had an increased caecal content of Salmonella when compared to the broiler groups [20]. However, the Barnevelders were able to clear the infection whereas the broilers even had a slight increase of Salmonella in the caecum. Furthermore, the Barnevelders also had the highest levels of anti-Salmonella antibodies [20]. In our experiments, the Barnevelders had the strongest antibody response against *C. jejuni*, and the lowest level of caecal colonization at 27 days, suggesting a general enhanced immune response towards colonizing bacteria in these chickens.

Previous exposure to pathogens may be another determining factor in the generation of anti-ganglioside antibodies following inoculation with *C. jejuni*. The immune system is shaped by continuous encounters with microbial flora. Passively transferred maternal anti-*C. jejuni* antibodies may have influenced the kinetics and strength of the anti-*C. jejuni* response in Barnevelders, leading to an earlier and stronger anti-ganglioside response [21].

We observed a large variation in antibody response and caecal colonization within chicken lines. This reflects the situation in humans [12]. The chicken lines used for these experiments are all outbred lines and thus there is considerable genetic variation between individual chickens of a single line. Studies into the influence of gene polymorphisms in immune response genes on *Salmonella* colonization of chickens found associations with multiple polymorphisms on colonization, even within groups of chickens [20]. Alternatively, differences in anti-ganglioside antibody formation may have been influenced by bacterial factors. Our observation that in some broiler groups, animals with high caecal bacterial counts have the strongest anti-glycolipid response suggests a threshold mechanism with respect to production of anti-glycolipid antibodies. Furthermore, transcription profiling has demonstrated a huge variation in expression of bacterial genes, including those associated with increased virulence [22]. Finally, differential expression of ganglioside mimics by *Campylobacter* cells during infection may also have been caused by phase variation in genes coding for enzymes involved in lipo-oligosaccharide synthesis [23].

Combined, these findings suggest that genetic differences within lines may be responsible for the variation in anti-ganglioside responses within groups. In this respect, a familial outbreak of *Campylobacter* diarrhoea with only one GBS case, illustrates that *Campylobacter* infections in genetically related human individuals can also have very different outcomes [12].

In one experiment the chickens developed neurological symptoms. We ruled out a concurrent infection with Marek's disease virus. Histopathological investigations of the nerves did not reveal any abnormalities, in contrast to one earlier study that described the presence of severe axonal degeneration in post-*C. jejuni* paralysed chickens [9]. Furthermore, there was no relation between the presence of neurological symptoms and circulating anti-ganglioside antibodies. Therefore, we can not completely rule out that the symptoms have been induced by *C. jejuni* induced anti-ganglioside antibodies but this seems highly unlikely.

In conclusion, we have shown that natural colonization with a GBS-associated *C. jejuni* strain is able to induce specific cross-reactive anti-LOS/ganglioside antibodies. Furthermore, the qualitative and quantitative differences between and within groups of chickens emphasize host-dependent factors in the generation of anti-ganglioside antibodies following *C. jejuni* inoculation and make the chicken infection model suited for studying the pathogenesis of GBS following *C. jejuni* infection.
